# Association of anterior crossbite and open bite with the number of remaining teeth: A cross-sectional study from the Tohoku medical megabank cohort

**DOI:** 10.1007/s00784-025-06715-5

**Published:** 2026-01-08

**Authors:** Kento Numazaki, Toru Tamahara, Takamasa Komiyama, Takako Numazaki, Maki Goto, Ritsuko Shimizu, Itaru Mizoguchi, Kaoru Igarashi, Hiroyasu Kanetaka

**Affiliations:** 1 Division of Orthodontics and Dentofacial Orthopedics, Tohoku University Graduate School of Dentistry, 4-1 Seiryo-machi, Aoba-ku, Sendai, Miyagi 980-8575 Japan; 2 Tohoku Medical Megabank Organization, Tohoku University, Sendai, 980-0873 Japan; 3 Division of Aging and Geriatric Dentistry, Department of Rehabilitation Dentistry, Tohoku University Graduate School of Dentistry, Sendai, 980-8575 Japan; 4 Private practice, Sendai, Japan; 5 Division of Craniofacial Anomalies, Tohoku University Graduate School of Dentistry, Sendai , 980-8575 Japan; 6 Division of Advanced Dental Science and Technology, Graduate School of Biomedical Engineering, Tohoku University, Sendai, 980-8579 Japan

**Keywords:** Crossbite, Open bite, Tooth loss, Orthodontics, Cross-Sectional studies, Epidemiology

## Abstract

**Objectives:**

Malocclusion, particularly anterior crossbite and open bite, contributes to abnormal occlusal stress distribution, potentially leading to tooth loss. We examined the association between anterior malocclusions and tooth loss–related outcomes in a large-scale, community-based sample of Japanese adults.

**Materials and methods:**

This cross-sectional study targeted 17,349 participants aged ≥ 40 years from the Tohoku Medical Megabank Cohort Study (2013–2017). Based on overjet and overbite, participants were classified into normal occlusion (*n* = 16,790), anterior open bite (*n* = 177), anterior crossbite (*n* = 348), and combined malocclusion (*n* = 34). Outcomes included ≤ 19 remaining natural teeth and posterior tooth loss. Adjusted prevalence ratios (PRs) and 95% confidence intervals (CIs) were calculated using modified Poisson regression models.

**Results:**

The crossbite group demonstrated a higher prevalence of ≤ 19 remaining teeth (PR, 1.48; 95% CI, 1.04–2.10) and posterior tooth loss (PR, 1.14; 95% CI, 1.07–1.20) than the normal group; the open bite group exhibited a lower prevalence of posterior tooth loss (PR, 0.79; 95% CI, 0.69–0.90). Heatmap analysis revealed lower molar tooth retention in the crossbite group and higher retention in the open bite group.

**Conclusions:**

Anterior crossbite is associated with increased posterior tooth loss, whereas open bite shows a weaker association. These findings suggest that specific anterior malocclusions may be linked to reduced tooth retention.

**Clinical relevance:**

Identification of anterior malocclusion types, particularly anterior crossbite, may help identify individuals at higher risk for tooth loss. Orthodontic management of anterior crossbite might support posterior dentition preservation and long-term oral function.

**Supplementary information:**

The online version contains supplementary material available at 10.1007/s00784-025-06715-5.

## Introduction

Malocclusion is an occlusal abnormality that can affect oral health -related quality of life [1]. In particular, anterior crossbite and anterior open bite may interfere with proper anterior occlusal contact, resulting in impaired masticatory function and localized concentration of occlusal forces on the posterior teeth. Anterior crossbite has also been associated with deteriorating periodontal conditions [2], and within the same population, occlusal trauma reportedly further aggravated the periodontal status of posterior teeth [3]. These findings suggest that the interplay between occlusal abnormalities and periodontal conditions may increase the risk of tooth loss.

Tooth loss becomes more common after age 40 [4], primarily due to caries and periodontal disease. Recently, malocclusion has emerged as a potential risk factor. Previous studies have reported significant associations between tooth loss and conditions such as an overjet ≥ 3 mm, deep bite with gingival contact, and posterior cusp-to-cusp bite [5]. Studies based on the Eichner classification have shown that reduced posterior occlusal support increases the risk of posterior tooth loss and may also affect the anterior teeth [6, 7]. Additionally, among older adults who had ≥ 20 remaining teeth at 80 years of age, none exhibited anterior crossbite or open bite [8], further supporting the epidemiological link between malocclusions and tooth loss. In the present study, individuals with < 20 remaining teeth were defined as having substantial tooth loss. This threshold has been widely used because the presence of < 20 teeth has been significantly associated with various adverse health outcomes, including poor nutritional intake [9], increased frailty [10], and cognitive decline [11]. These findings reaffirm the importance of tooth retention for extending healthy life expectancy.

Currently, large-scale epidemiological studies assessing the relationship between anterior crossbite or open bite and tooth loss are scarce, particularly among adults. Moreover, few studies have evaluated the location of tooth loss in relation to specific types of malocclusions. Clarifying the association between malocclusion and tooth loss could provide a scientific basis for the contribution of orthodontic treatment to tooth preservation and hold important implications for oral health policy and preventive dentistry.

This study aimed to investigate the association between malocclusion and the number of remaining teeth in adults aged ≥ 40 years to elucidate the impact of malocclusion on tooth loss.

## Materials and methods

### Study design and population

**Fig. 1:**
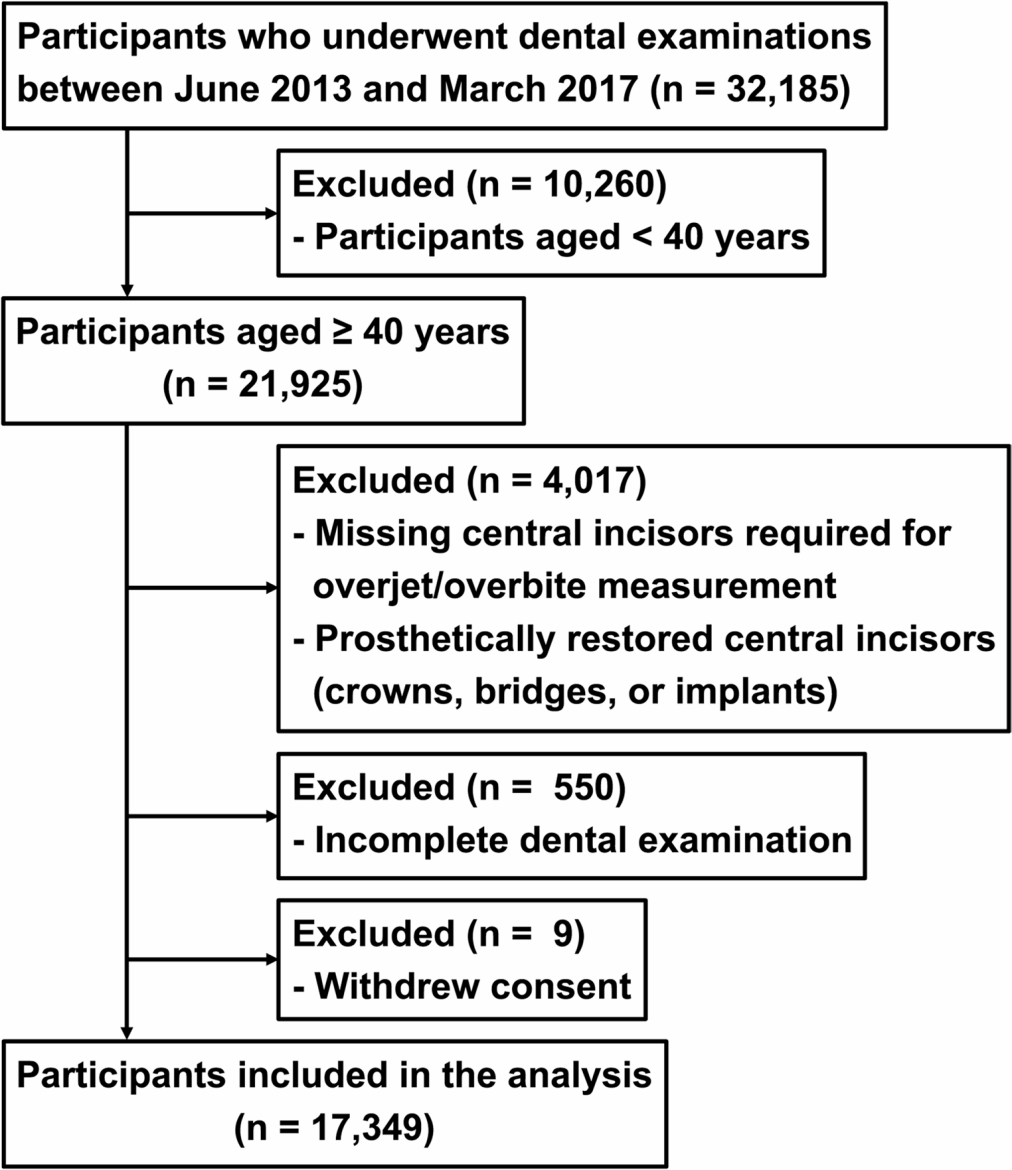
Flow diagram showing the recruitment of study participants

This cross-sectional study used baseline data from the Tohoku Medical Megabank Cohort Study (TMM Cohort Study), a cohort survey of the Tohoku Medical Megabank (TMM) Project conducted in Miyagi Prefecture, Japan, from 2013. The TMM Project was launched to support the recovery of medical care in areas severely affected by the Great East Japan Earthquake on March 11, 2011 [12]. Among 37,575 participants aged ≥ 20 years registered in the TMM Cohort Study, 21,925 participants aged ≥ 40 years who underwent dental examinations (June 2013–May 2017) were included, as tooth loss increases markedly after the age of 40 [4, 13]. Participants were excluded if they had missing or prosthetically restored maxillary and mandibular central incisors (including crowns, bridges, or implants), which made accurate measurement of overjet and overbite infeasible; if their dental examination data were incomplete; or if they had withdrawn consent. In total, 17,349 participants were included in the final analysis (Fig. 1).

Participants aged ≥ 40 years from the Tohoku Medical Megabank Cohort Study (2013–2017) were included. Individuals with missing or prosthetically restored maxillary or mandibular central incisors, incomplete dental examination, or withdrawn consent were excluded, resulting in a final analytic sample of 17,349 participants.

This study followed the Declaration of Helsinki (1964) and comparable ethical standards. The study protocol was approved by the Research Ethics Committee of the Graduate School of Dentistry, Tohoku University (approval number: 37703). Written informed consent was waived in accordance with institutional guidelines, and participants were provided with an opt-out option to ensure that their rights and autonomy were respected. The study followed the Strengthening the Reporting of Observational Studies in Epidemiology (STROBE) guidelines for reporting cross-sectional studies.

### Dental examinations

Dental examinations were performed by dentists who received specific instructions during orientation meetings and on-the-job training prior to participation in the oral examination program [12]. A supervising dentist provided initial on-the-job training and conducted periodic follow-up sessions and quality assessments over several months. The number of remaining teeth and overjet/overbite values were assessed.

Overjet and overbite were measured using a periodontal probe (Code No. 28107, YDM Co., Tokyo, Japan). The distance (in mm) between the incisal edges of the maxillary and mandibular right central incisors in the occlusal position was measured. When the right central incisor was missing, the left central incisor was used instead. For participants with removable dentures, measurements were conducted with the dentures in place. Participants were classified into four groups based on their overjet and overbite values:

- Normal overjet and overbite group (normal group): overjet ≥ 0 mm and overbite ≥ 0 mm.
- Anterior open bite group (open bite group): overjet ≥ 0 mm and overbite < 0 mm.
- Anterior crossbite group (crossbite group): overjet < 0 mm and overbite ≥ 0 mm.
- Combined open and crossbite group (combined group): overjet < 0 mm and overbite < 0 mm.

The number of remaining teeth, including retained roots, was recorded, and participants were categorized into two groups: those with ≥ 20 teeth and those with ≤ 19 teeth. Posterior tooth loss was evaluated as a binary variable (presence or absence). Dental caries were recorded based on their presence at the time of examination. The severity of periodontal disease was categorized based on the proportion of teeth, with a probing depth ≥ 4 mm: healthy (0%), mild (> 0%–≤20%), moderate (> 20%–≤50%), and severe (> 50%) [14]. Oral hygiene status was categorized as mild (no plaque or plaque in the form of a thin film), moderate (visible plaque), or severe (dense plaque) based on the degree of plaque accumulation on the remaining teeth.

### Covariates

Based on previous studies, the following variables were selected as potential confounders related to tooth loss: body mass index (BMI) [15], hypertension [16], diabetes [17], smoking status [18], alcohol consumption [19], and educational attainment [13]. BMI was classified into three categories: underweight (< 18.5 kg/m²), normal weight (18.5–25 kg/m²), and obese (≥ 25 kg/m²) [20]. Hypertension was defined as self-reported treatment for hypertension or measured systolic blood pressure ≥ 140 mmHg or diastolic blood pressure ≥ 90 mmHg, based on the guidelines of the Japanese Society of Hypertension [21]. Diabetes was defined as self-reported treatment or HbA1c ≥ 6.5%. Smoking, drinking, and educational status were assessed using a self-administered questionnaire. Smoking status was categorized as current smoker, former smoker (defined as having smoked ≥ 100 cigarettes in their lifetime), or never smoker. Alcohol consumption was classified as current, former, or never. Educational attainment (≤ 12 years of education) was determined based on whether the participant had graduated from high school.

### Statistical analysis

For baseline characteristics, continuous variables are described as mean and standard deviation, and categorical variables as percentages. Intergroup comparisons were made using Kruskal–Wallis tests and Fisher’s exact tests. To evaluate the association between malocclusion and outcomes (tooth loss or posterior tooth loss), modified Poisson regression analysis was employed to estimate the prevalence ratios (PRs) and 95% confidence intervals (CIs). Three models were applied as follows: a univariate model (Model 1); a model adjusted for age and sex (Model 2); and a model adjusted for age, sex, BMI, active caries, periodontal disease severity, oral hygiene status, hypertension, diabetes, smoking status, alcohol consumption, and educational attainment (Model 3). The main results presented in the manuscript are based on Model 3. Sensitivity analysis was conducted using Poisson regression models to assess the association between malocclusion and tooth loss, with the number of remaining teeth as a continuous variable. The results are presented as rate ratios (RRs).

All statistical analyses were conducted using SAS version 9.4 (SAS Institute Inc., Cary, NC, USA). Two-sided p-values < 0.05 were considered statistically significant. Missing data were handled to minimize data loss and maintain statistical power. Missing covariate values (e.g., periodontitis, plaque accumulation, BMI, hypertension, diabetes, smoking, alcohol consumption, and education) were categorized as independent “missing” groups. Model diagnostics were used to confirm an appropriate model fit with no multicollinearity.

### Heatmap of remaining teeth prevalence

To compare the distribution of remaining teeth across the four malocclusion groups, heatmaps were generated based on the prevalence of each tooth. Prevalence was calculated as the proportion of participants who retained each specific tooth. Teeth with 100% prevalence were shown in white, while those with ≤ 40% were displayed in black; intermediate values were represented using a gradient scale. The heatmaps were stratified by age groups: 40–49, 50–59, 60–69, and ≥ 70 years.

## Results

### Association between malocclusion and the number of remaining teeth

A total of 17,349 participants aged ≥ 40 years were included in the analysis. The mean age was 59.3 years, and 68.1% were female. The prevalence of malocclusion types was as follows: 16,790 were classified into the normal group, 177 into the open bite group, 348 into the crossbite group, and 34 into the combined group. Table 1 shows the baseline characteristics of each malocclusion group. Significant intergroup differences were observed in age, sex, oral hygiene status, BMI, alcohol consumption, number of remaining teeth, and presence of posterior tooth loss.

**Table 1:** Baseline characteristics according to the classification of malocclusion

		Classification of malocclusion				
Variable	Overall	Normal	Open bite	Crossbite	Combined	*P* value
	(*N* = 17,349)	(*n* = 16,790)	(*n* = 177)	(*n* = 348)	(*n* = 34)	
Age, mean ± SD	59.27 ± 9.7	59.39 ± 9.7	55.51 ± 9.7	55.77 ± 9.4	52.06 ± 9.5	< 0.001
Sex, %						0.004
Male	31.9	31.8	26.6	35.6	55.9	
Female	68.1	68.2	73.4	64.4	44.1	
Active caries, %						0.273
Yes	28.9	28.8	29.4	32.5	38.2	
No	71.1	71.2	70.6	67.5	61.8	
Periodontitis severity, %						0.096
Healthy	34.2	34.2	26.0	36.8	26.5	
Mild	42.4	42.4	44.1	37.1	41.2	
Moderate	17.5	17.4	22.0	19.0	26.5	
Severe	5.7	5.6	7.3	6.3	5.9	
Missing	0.3	0.3	0.6	0.9	0.0	
Plaque accumulation, %						0.002
Mild	77.0	77.2	74.0	70.7	76.5	
Moderate	19.4	19.3	17.5	24.4	20.6	
Severe	3.4	3.3	7.9	4.9	2.9	
Missing	0.2	0.2	0.6	0.0	0.0	
BMI, %						0.011
< 18.5	6.5	6.5	5.6	4.9	8.8	
18.5–25	68.0	68.2	65.0	62.6	55.9	
> 25	24.8	24.6	27.7	31.0	32.4	
Missing	0.7	0.7	1.7	1.4	2.9	
Hypertension, %						0.837
Yes	36.6	36.6	33.9	37.4	32.4	
No	63.1	63.1	66.1	62.4	67.6	
Missing	0.3	0.3	0.0	0.3	0.0	
Diabetes, %						0.211
Yes	5.9	5.9	5.6	8.3	8.8	
No	92.7	92.7	91.5	90.5	91.2	
Missing	1.4	1.4	2.8	1.1	0.0	
Smoking, %						0.460
Current	9.5	9.3	13.0	12.4	14.7	
Former	23.4	23.4	22.0	23.6	26.5	
Never	61.0	61.1	59.9	57.8	55.9	
Missing	6.2	6.2	5.1	6.3	2.9	
Alcohol consumption, %						0.012
Current	54.6	54.4	52.5	63.2	67.6	
Former	5.9	5.9	7.3	5.5	5.9	
Never	39.1	39.3	40.1	30.7	23.5	
Missing	0.4	0.4	0.0	0.6	2.9	
Educational attainment, %						0.650
≤ 12 years	55.1	55.1	50.3	56.9	47.1	
> 12 years	41.8	41.8	45.8	40.8	50.0	
Missing	3.1	3.1	4.0	2.3	2.9	
Number of teeth, %						0.026
≤ 19	6.8	6.8	2.3	8.6	2.9	
≥ 20	93.2	93.2	97.7	91.4	97.1	
Absence of molars, %						< 0.001
Yes	71.4	71.5	54.2	75.9	67.6	
No	28.6	28.5	45.8	24.1	32.4	

*P* values were obtained using Kruskal–Wallis tests for continuous variables and Fisher’s exact tests for categorical variables

*BMI*body mass index,* SD* standard deviation

To examine the association between malocclusions and the number of remaining teeth, we conducted modified Poisson regression analysis with ≤ 19 remaining natural teeth as the outcome (Table 2). In Model 1, the open bite group had a lower proportion with ≤ 19 remaining teeth than the normal group (PR, 0.33; 95% CI, 0.13–0.87). In Models 2–3, the crossbite group showed higher proportions (Model 2: PR, 1.69; 95% CI, 1.19–2.39; Model 3: PR, 1.48; 95% CI, 1.04–2.10). The combined group showed no significant differences. In the sensitivity analysis, Poisson regression models with the number of remaining teeth as a count variable were used (Online Resource 1). Anterior crossbite was significantly associated with fewer remaining teeth (RR, 0.98; 95% CI, 0.96–1.00), while open bite showed a weak positive association (RR, 1.04; 95% CI, 1.01–1.07), which was not statistically significant. No significant association was observed in the combined group.

**Table 2:** Modified Poisson regression model for the association between malocclusion and tooth loss outcomes (*n* = 17,349)

Variables	Participants, *n*	Outcome: ≤ 19 remaining teeth			Outcome: Posterior tooth loss		
		Model 1	Model 2	Model 3	Model 1	Model 2	Model 3
		PR (95% CI), *P* value					
Normal	16,790	1.00 (Reference)	1.00 (Reference)	1.00 (Reference)	1.00 (Reference)	1.00 (Reference)	1.00 (Reference)
Open bite	177	0.33 (0.13–0.87), 0.026	0.44 (0.16–1.15), 0.093	0.43 (0.16–1.14), 0.088	0.76 (0.66–0.87), < 0.001	0.82 (0.72–0.93), 0.003	0.79 (0.69–0.90), < 0.001
Crossbite	348	1.26 (0.89–1.79), 0.187	1.69 (1.19–2.39), 0.003	1.48 (1.04–2.10), 0.029	1.06 (1.00–1.13), 0.055	1.15 (1.08–1.21), < 0.001	1.14 (1.07–1.20), < 0.001
Combined	34	0.43 (0.06–2.97), 0.393	0.77 (0.11–5.48), 0.795	0.84 (0.12–5.94), 0.862	0.95 (0.75–1.19), 0.638	1.11 (0.90–1.38), 0.325	1.10 (0.88–1.38), 0.386

Model 1: crude; Model 2: adjusted for age and sex; Model 3: adjusted for age, sex, active caries, periodontitis severity, plaque accumulation, BMI, hypertension, diabetes, smoking, alcohol consumption, education

*BMI *body mass index, *CI* confidence interval,* PR* prevalence ratio

### Association between malocclusion and posterior tooth loss

Modified Poisson regression analyses were also conducted to assess the association between malocclusions and posterior tooth loss (Table 2). In the open bite group, the proportion of participants with posterior tooth loss was consistently lower than in the normal group across all models (Model 1: PR, 0.76; 95% CI, 0.66–0.87; Model 2: PR, 0.82; 95% CI, 0.72–0.93; Model 3: PR, 0.79; 95% CI, 0.69–0.90). In the crossbite group, the PRs were higher in Model 2 (PR, 1.15; 95% CI, 1.08–1.21) and Model 3 (PR, 1.14; 95% CI, 1.07–1.20) than in the normal group. No significant differences were observed between the combined and normal groups across all models. In the sensitivity analysis, Poisson regression was also applied to evaluate the association between malocclusion and the absence of molars (Online Resource 2). Open bite remained significantly associated with a lower prevalence of molar loss (RR, 0.65; 95% CI, 0.57–0.73), while anterior crossbite was associated with a higher prevalence of molar loss (RR, 1.12; 95% CI, 1.00–1.27). These findings further support the robustness of the main analysis.

### Age-specific patterns of tooth loss

#### Age-specific patterns of tooth loss

**Fig. 2:**
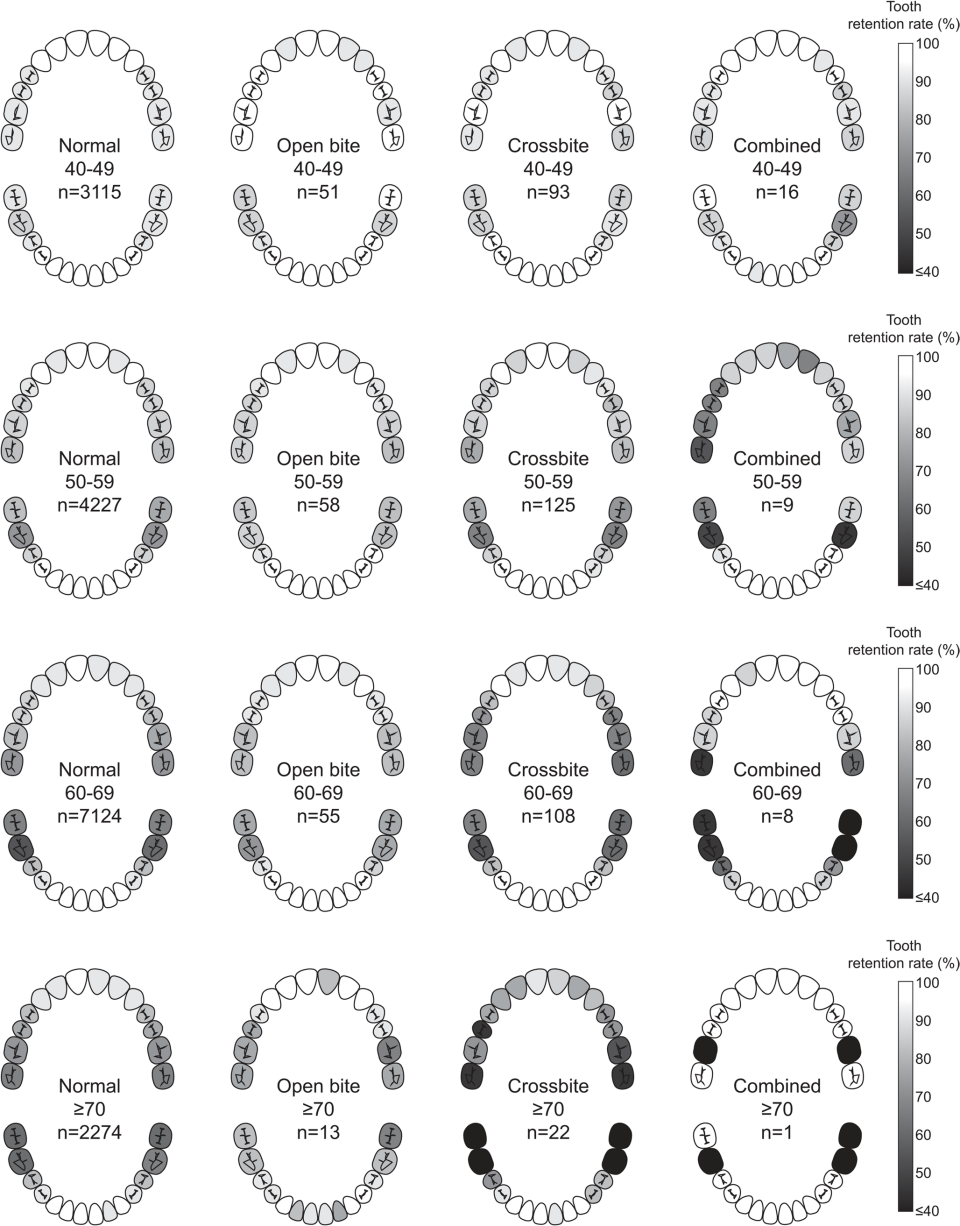
Heatmaps showing age-specific tooth retention by malocclusion group

Figure 2 shows heatmaps illustrating the age-specific distribution of remaining teeth according to malocclusion type. These heatmaps are descriptive visualizations that complement the regression results presented in Table 2 and do not represent statistical testing.

In participants in their 40 s, the retention rate of all teeth was generally ≥ 85% across all groups. In the 50 s, the mandibular first molar retention rate in the crossbite group (66%) was lower than in the normal (70%) and open bite (80%) groups. In the 60 s, the retention of maxillary and mandibular molars in the crossbite group declined to < 70%, and among participants aged ≥ 70 years, molar retention in the crossbite group was the lowest (≤ 45%). These findings suggest that posterior tooth loss was more likely to occur with increasing age in the crossbite group. In contrast, the open bite group maintained ≥ 70% retention of mandibular molars and ≥ 80% for other teeth in their 60 s, with most teeth showing ≥ 75% retention in participants aged ≥ 70 years. These findings indicate that open bite may be associated with a relatively lower risk of posterior tooth loss.

Tooth-specific prevalence is presented in each panel, showing the proportion of participants retaining a given tooth. Grayscale indicates tooth retention (white = 100% retention; black = ≤ 40% retention). Heatmaps are stratified by age group (40–49, 50–59, 60–69, ≥ 70 years) and malocclusion type (normal, open bite, crossbite, combined). Missing teeth are indicated in darker shades. Higher retention corresponds to lighter tones.

## Discussion

This study examined the association between malocclusion and the number of remaining teeth and posterior tooth loss among adults aged ≥ 40 years. After adjusting for age, sex, BMI, active caries, periodontal disease severity, oral hygiene status, hypertension, diabetes, smoking, alcohol consumption, and educational attainment, crossbite was associated with a reduced number of remaining teeth, particularly due to posterior tooth loss. In contrast, open bite was associated with a lower prevalence of posterior tooth loss. These findings were consistent with sensitivity analyses, supporting the robustness of the associations. To our knowledge, this is the first study to demonstrate an association between anterior crossbite and the number of remaining teeth using data from a community-based study. Because anterior crossbite and open bite are relatively uncommon in the general population, large-scale epidemiological data are essential for verifying their associations with tooth loss. Our study addresses this gap using a well-powered dataset.

Our findings showed a significant reduction in the number of remaining teeth in the crossbite group, particularly due to posterior tooth loss, compared with the normal group. Heatmap analysis also revealed notable losses in the mandibular and maxillary molars, as well as the maxillary second premolars. These results are consistent with previous case reports of middle-aged patients with anterior crossbite presenting with multiple missing posterior teeth [22, 23].

Patients with anterior crossbite often lack anterior or canine guidance during protrusive and lateral movements, resulting in continued contact of posterior teeth during mandibular excursions [24]. This condition subjects the posterior teeth to lateral occlusal forces, which may contribute to increased occlusal load and potential tooth loss, especially in individuals with compromised periodontal health. Indeed, previous studies have reported that occlusal trauma can exacerbate the destruction of periodontal tissues and contribute to tooth loss [25]. Furthermore, individuals with anterior crossbite often present with a Class III molar relationship, in which the maxillary second premolars make sliding contact with the mandibular first and second molars, potentially imposing excessive occlusal forces on these teeth and resulting in their loss. Despite adjustment for caries, periodontitis severity, and plaque accumulation, crossbite remained associated with tooth loss, indicating an independent effect; it may also exacerbate disease via increased occlusal stress on periodontal tissues. Therefore, both independent and interactive effects should be considered.

In contrast, the open bite group showed a significantly lower prevalence of posterior tooth loss and higher tooth retention rates than the other groups. This finding aligns with previous findings suggesting that a mild anterior open bite (1–2 mm) may have a protective effect against tooth loss [5]. Although patients with an anterior open bite also tend to lack anterior guidance, which may increase posterior contact during mandibular movement, several studies have reported that such individuals exhibit weaker occlusal forces [26, 27]. In addition, limited condylar movement has been reported in open bite cases [26], which, together with weaker occlusal forces, may reduce lateral loading on posterior teeth and mitigate the risk of occlusal trauma. Notably, the normal group in our study included participants with excessive overjet and overbite, which have been reported to negatively affect periodontal conditions [28]. Therefore, the risk of posterior tooth loss in the open bite group may have been underestimated in this study. Thus, the apparent “protective” association of open bite should be interpreted cautiously; further studies with detailed occlusal assessments are warranted. Nevertheless, there is limited evidence suggesting that an anterior open bite contributes to the prevention of posterior tooth loss. Therefore, further research is needed to elucidate the underlying mechanisms.

In the combined group, there were no significant differences in the number of remaining teeth or the prevalence of posterior tooth loss compared with those in the normal group. The heatmap showed a slight reduction in mandibular molar retention; however, the maxillary molars and second premolars were retained at higher rates than in the crossbite group. On the other hand, overall tooth retention in the combined group was lower than that in the open bite group. These findings suggest that the increased risk of tooth loss due to an anterior crossbite and the potential protective effect of an open bite may have interacted in a way that attenuated observable differences in the combined group. However, the small sample size of the combined group limited the statistical power to draw definitive conclusions; thus, further studies are needed.

This study has several limitations. First, since this was a cross-sectional study, a causal relationship between malocclusion and tooth loss could not be established. Thus, future longitudinal studies are needed to further investigate this relationship. Second, the study population was limited to residents in specific regions of Japan, which may limit generalizability to other populations. Nonetheless, this large-scale dataset was derived from a community-based cohort and is considered representative of the regional population. Third, markedly uneven sample sizes limited the statistical power and reliability of subgroup comparisons; results for the combined group should be interpreted with caution; nonetheless, the main findings relied on adequately powered groups. Fourth, anterior crossbite has skeletal and dentoalveolar causes; cephalometric radiographs were unavailable; thus, skeletal morphology could not be evaluated. In addition, overjet and overbite were assessed as positive or negative values rather than in millimeters since this was a community-based examinations. Future studies using dental casts or cephalometric imaging are needed for a more precise classification and evaluation of the relationship between skeletal morphology and tooth loss. Finally, including only participants with intact central incisors may have overestimated incisor retention; anterior tooth loss could not be evaluated. Longitudinal studies are needed to clarify whether anterior crossbite predisposes to anterior tooth loss.

## Conclusions

These findings suggest that the type of malocclusion is associated with tooth loss and supports the potential role of orthodontic treatment in tooth preservation. Early diagnosis and management of anterior crossbite, and assessing overjet and overbite in community examinations, may help prevent tooth loss.

## Supplementary information

Below is the link to the electronic supplementary material.

Supplementary File 1 (PDF 75.5 KB)

## Data Availability

The datasets analyzed during the current study are not publicly available due to participant privacy protection.
