# Supplementary material for: Association of anterior crossbite and open bite with the number of remaining teeth: A cross-sectional study from the Tohoku medical megabank cohort
Source: Clin Oral Investig. 2026 Jan 8;30(1):43. doi: 10.1007/s00784-025-06715-5 (PMC12779704; doi:10.1007/s00784-025-06715-5)
Supplement: Supplementary file 1 — (PDF 75.5 KB) [file 784_2025_6715_MOESM1_ESM.pdf]

**Online Resource 1.** Poisson regression models evaluating the association between anterior malocclusion and the number of present teeth as a continuous outcome.

| Variables | Participants, n | Model 1 | Model 2 | Model 3 |
|---|---|---|---|---|
| | | RR (95% CI), *P* value | | |
| Normal | 16,790 | 1.00 (Reference) | 1.00 (Reference) | 1.00 (Reference) |
| Open bite | 177 | 1.05 (1.00–1.01), <0.001 | 1.04 (1.01–1.07), 0.017 | 1.04 (1.01–1.07), 0.081 |
| Crossbite | 348 | 0.99 (0.97–1.00), 0.328 | 0.97 (0.95–0.995), 0.016 | 0.98 (0.96–1.00), 0.043 |
| Combined | 34 | 1.00 (0.94–1.07), 0.89 | 0.97 (0.91–1.04), 0.409 | 0.97 (0.91–1.04), 0.457 |

Model 1: crude; Model 2: adjusted for age and sex; Model 3: adjusted for age, sex, active caries, periodontitis severity, plaque accumulation, BMI, hypertension, diabetes, smoking, alcohol consumption, education

*BMI*, body mass index; *CI*, confidence interval; *RR*, rate ratio

**Article title:** Association of anterior crossbite and open bite with the number of remaining teeth: a cross-sectional analysis from the Tohoku Medical Megabank Cohort Study

**Journal name:** Clinical Oral Investigations

**Authors:** Kento Numazaki, Toru Tamahara, Takamasa Komiyama, Takako Numazaki, Maki Goto, Ritsuko Shimizu, Itaru Mizoguchi, Kaoru Igarashi, Hiroyasu Kanetaka

**Corresponding author:**

Kento Numazaki: Division of Orthodontics and Dentofacial Orthopedics, Tohoku University Graduate School of Dentistry, Email address: kento.numazaki.d7@tohoku.ac.jp

**Online Resource 2.** Poisson regression models evaluating the association between anterior malocclusion and the number of missing molars as a continuous outcome.

| Variables | Participants, n | Model 1 | Model 2 | Model 3 |
|---|---|---|---|---|
| | | RR (95% CI), *P* value | | |
| Normal | 16,790 | 1.00 (Reference) | 1.00 (Reference) | 1.00 (Reference) |
| Open bite | 177 | 0.58 (0.52–0.65), <0.001 | 0.68 (0.60–0.76), <0.001 | 0.65 (0.57–0.73), <0.001 |
| Crossbite | 348 | 1.06 (1.00–1.13), 0.061 | 1.24 (1.17–1.32), <0.001 | 1.12 (1.00–1.27), <0.001 |
| Combined | 34 | 1.00 (0.81–1.22), 0.963 | 1.37 (1.12–1.68), 0.002 | 1.35 (1.09–1.66), 0.005 |

Model 1: crude; Model 2: adjusted for age and sex; Model 3: adjusted for age, sex, active caries, periodontitis severity, plaque accumulation, BMI, hypertension, diabetes, smoking, alcohol consumption, education

*BMI*, body mass index; *CI*, confidence interval; *RR*, rate ratio

**Article title:** Association of anterior crossbite and open bite with the number of remaining teeth: a cross-sectional analysis from the Tohoku Medical Megabank Cohort Study

**Journal name:** Clinical Oral Investigations

**Authors:** Kento Numazaki, Toru Tamahara, Takamasa Komiyama, Takako Numazaki, Maki Goto, Ritsuko Shimizu, Itaru Mizoguchi, Kaoru Igarashi, Hiroyasu Kanetaka

**Corresponding author:**

Kento Numazaki: Division of Orthodontics and Dentofacial Orthopedics, Tohoku University Graduate School of Dentistry, Email address: kento.numazaki.d7@tohoku.ac.jp
